# Topologically driven Rabi-oscillating interference dislocation

**DOI:** 10.1515/nanoph-2022-0108

**Published:** 2022-05-09

**Authors:** Amir Rahmani, David Colas, Nina Voronova, Kazem Jamshidi-Ghaleh, Lorenzo Dominici, Fabrice P. Laussy

**Affiliations:** Department of Physics, Azarbaijan Shahid Madani University, Tabriz, Iran; Aix Marseille Université, CNRS, Centrale Marseille, LMA UMR 7031 Marseille, France; National Research Nuclear University MEPhI (Moscow Engineering Physics Institute), 115409 Moscow, Russia; Russian Quantum Center, Skolkovo Innovation City, 121205 Moscow, Russia; CNR NANOTEC, Istituto di Nanotecnologia, Via Monteroni, 73100 Lecce, Italy; Faculty of Science and Engineering, University of Wolverhampton, Wulfruna Street, WV1 1LY Wolverhampton, UK

**Keywords:** exciton-polariton, interference dislocation, linear momentum, orbital angular momentum, self-interfering wavepacket

## Abstract

Quantum vortices are the quantized version of classical vortices. Their center is a phase singularity or vortex core around which the flow of particles as a whole circulates and is typical in superfluids, condensates and optical fields. However, the exploration of the motion of the phase singularities in coherently-coupled systems is still underway. We theoretically analyze the propagation of an interference dislocation in the regime of strong coupling between light and matter, with strong mass imbalance, corresponding to the case of microcavity exciton–polaritons. To this end, we utilize combinations of vortex and tightly focused Gaussian beams, which are introduced through resonant pulsed pumping. We show that a dislocation originates from self-interference fringes, due to the non-parabolic dispersion of polaritons combined with moving Rabi-oscillating vortices. The morphology of singularities is analyzed in the Poincaré space for the pseudospin associated to the polariton states. The resulting beam carries orbital angular momentum with decaying oscillations due to the loss of spatial overlap between the normal modes of the polariton system.

## 1 Introduction

Many branches of Physics deal with phenomena involving superposition of two or more waves. For example, in optics, fringe pattrens are of a great interest, in particular the points of zero density, where the phase is undefined (or singular). Even in the absence of external control, the simplest-structured phases can result in exotic objects; a familiar example is given by the spiraling wavefront, typical in many so-called quantum fluids [1], [2], [3], [4], [5], [6], which is known as a quantum vortex state. This is described mathematically by the azimuthal phase factor e^i*lφ*^ where *l* is the winding number (or topological charge) denoting the number of twists around a region with null density. In this region, the phase is singular or indeterminate. It is noteworthy that this spatially localized point-like entity does not violate uncertainty relations, due to the phase singularity representing also a divergence of its gradient which maps the local momentum of particles. This is also balanced by the zero density at such a point. However, there exists a field amplitude in the rest of the space and so a density current winding around the singularity, implying the existence of orbital angular momentum (OAM).

**Figure 1:**
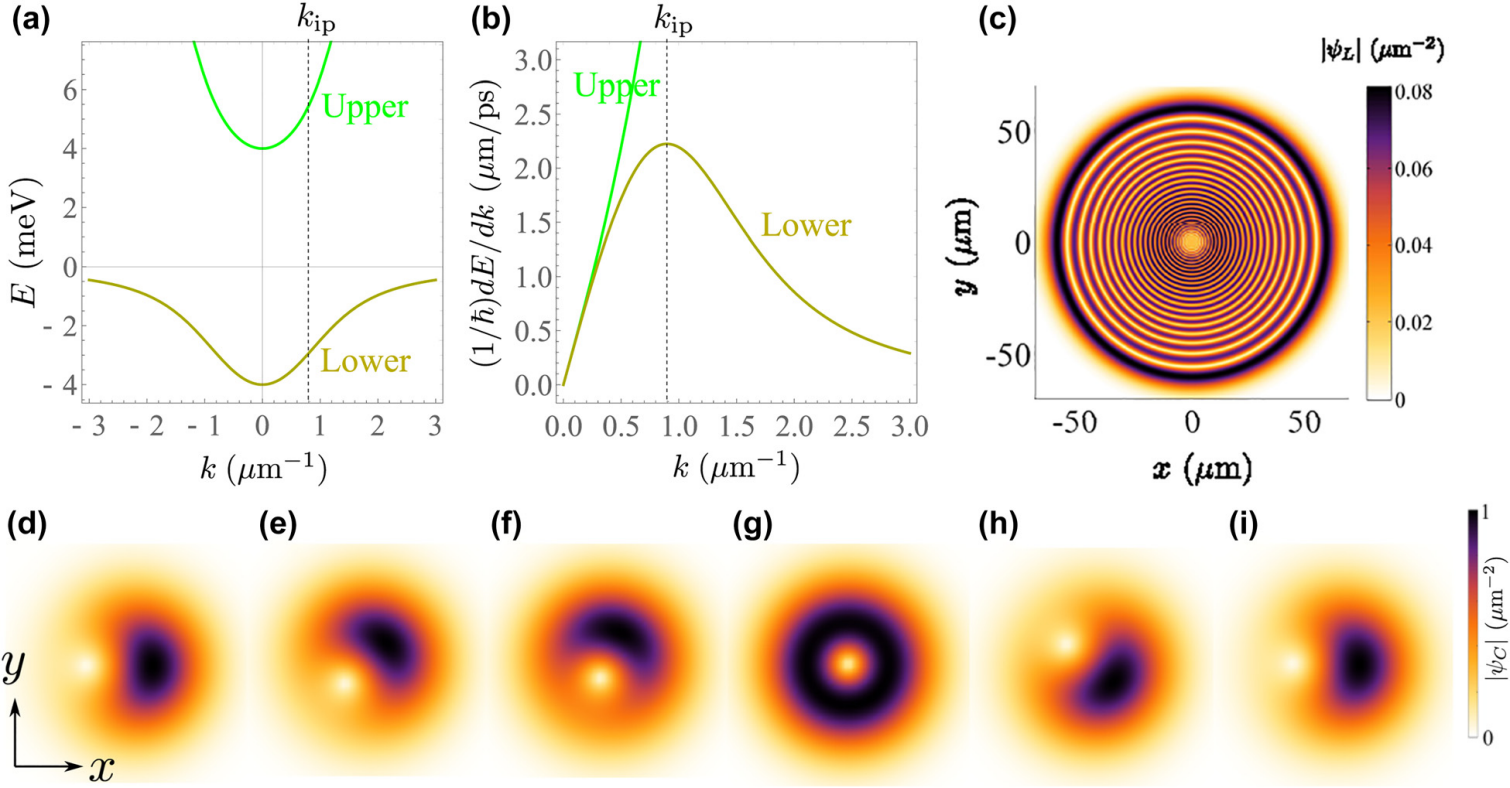
Background concepts. (a) Branches of exciton–polariton dispersion; the lower polariton dispersion, shown in dark yellow, deviates from the parabolic shape for *k* > *k*_ip_. The upper branch, shown in green, is always almost parabolic. Considering the lower branch, its concavity is upward for *k* > *k*_ip_ and is downward for *k* < *k*_ip_, and as a consequence *k*_ip_ is an inflection point. (b) The derivative of energy (*E*) with respect to wavenumber (*k*) shows a local maximum at the inflection point *k*_ip_, so the lower polariton packet is slower beyond *k*_ip_. (c) Example of self-interfering wavepacket for the lower polariton (*ψ*_L_), generated from a 1.5 μm wide initial state and after 200 ps of diffusion. This regime is reached by exciting with a momentum width extending over the inflection point of the lower branch, that is by a spatially tight beam, where the field interferes with itself. (d–i) Rabi oscillating  motion of a displaced vortex as seen in the photon component of the exciton–polariton field. The vortex core (the point of zero density) makes an entire counter-clockwise rotation during a Rabi cycle.. Here the size of the packet is large enough large in real space so that the wavepacket never straddles the inflection point.

Going beyond the concept of a helical phase front of a standard quantum vortex, the next level is represented by helical-vortex wavepackets [7], in the sense that the vortex core and the center of mass together with the net transverse linear momentum (NTLM) can themselves circulate in time (Figure 1(d)–(i)). At the next step, even the expectation value of the vortex angular momentum varies in time. Such a feature can be introduced in different ways. We recently showed [8], [9], [10] that through sending retarded and shaped optical pulses, a binary quantum fluid (i.e., made of two coupled components) can display OAM oscillations even in the linear regime, associated to a varying distance of the vortex core from the packet’s center. A similar sequence of time delayed pulses has been used to shape nonlinear processes of high harmonic generation, resulting in a time-varying OAM [11]. Also, external potentials can generate rotating wavepackets; model examples are harmonic potential [9] and ring shaped potential [12, 13] where the angular content is constant, or spiraling vortices can emerge from the sudden switch of a continuous-wave ring beam [14].

Interesting dynamics can happen in the case of two-coupled fields with strong mass imbalance [8, 15]. An example of such binary fields is the exciton–polariton, or in short polariton, formed in an optical cavity [16]. In this structure, the emitted light of an optically active quantum-well exciton (bound state of electron-hole in semiconductors) interacts efficiently with a cavity mode of electromagnetic radiation inside the cavity. Hence, the excitations can be exchanged several times between the excitonic and photonic fields until either the exciton collapses (e.g. due to scattering) or the photon escapes the cavity (e.g. due to tunneling through the mirrors). This results in the strong coupling regime of light–matter interaction, where the *coherent* coupling of quantum-well exciton with the cavity mode dominates all relaxation processes [17, 18] and where new eigenstates are formed, namely the lower and upper polariton states. One key feature of such eigenstates is their dispersion relation. Typical polariton energy-momentum dispersions are shown in Figure 1(a). Due to the mass imbalance between the coupled photonic and excitonic fields, the resulting dressed states (upper and lower polariton states) have peculiar dispersions, in particular, the lower branch is parabolic in the vicinity of *k* = 0 and deviates qualitatively from the parabola beyond an inflection point, at which the dispersion’s curvature changes sign, i.e., ${\text{d}}^{2}E\left(k\right)/\text{d}k^{2}{|}_{k=k_{\text{ip}}}=0$ where *E* stands for energy. For a wavepacket excited in the parabolic region of the dispersion, one can expect the usual propagation and diffusion [19–21]. The dynamics is startling when embracing states that go beyond the inflection point, leading to the concept of self-interfering wavepacket (SIP) [15]. A state extended in momentum space can be realized with a sharp wavepacket in real space. A sufficiently small wavepacket in real space then contains momenta spanning below and above the inflection point. Because of the peculiar dispersion of the lower polariton, increasing the momentum above the inflection point results in a decrease of the associated group velocity, as illustrated in Figure 1(b). A sharp wavepacket in real space can thus possess high and low-momentum components propagating together with the same group velocities, hence overlapping and interfering in real space, which forms regions with high and low densities, i.e. ripples. An example of such a self-interfering wavepacket is shown in Figure 1(c). Indeed, the non-parabolicity of the lower polariton dispersion leads to an apparent back reflection of high momentum lower polariton modes, since the highest velocity is reached at the inflection point and decreases beyond. A similar effect can also be observed when starting with a finite (linear) central momentum (see Supplementary Material). This stems once again from the different momenta propagating with different group velocities inside the lower mode packet, reaching different distances at a given time, as has been also discussed in the case of X-wave packets [22, 23], as well as for the two lower and upper modes propagating with different speeds [10].

Beside their non-parabolic dispersion, polaritons exhibit Rabi oscillations, which once again come from the periodic exchange of excitations between excitons and photons in the strong coupling regime. Rabi oscillations are common in many areas of physics; one can find examples in solid state physics [24–26], in Josephson junction [27], in cold atoms [28] and in excitons [29, 30], among others. While exciton–polaritons are Rabi-oscillating, their associated states within the cavity are represented by a superposition of photon and exciton states, forming the upper and lower polaritons. If such a superposition is cancelled, e.g. when photons leak out of the microcavity due to the reflectors’ imperfection, the superposition falls into one of the initial bases. Hence, thanks to the photon leakage, we can detect these escaping photons and measure associated observables, such as linear or orbital angular momentum. An example of the composite dynamics which can be observed experimentally and formed by a displaced vortex periodically rotating with the Rabi oscillations, is shown in Figure 1(d)–(i). Such a configuration can be generated when pulses carrying orbital angular momentum are injected with a mutual delay into the cavity [8, 10].

Here, we study in a two-dimensional setting the resulting dynamics from the combination of a vortex beam, similar to one leading to the dynamics shown in panels (e–j), and a tightly focused Gaussian beam which leads to an SIP. We introduce a new type of moving dislocation that arises from vortices in the presence of Rabi oscillations in the regime of self-interference. Namely, we use pulses carrying a topological charge and of small enough size in real space for their momenta to go beyond the inflection point (*k*_ip_), thereby quickly expanding and interfering in a highly structured way, in addition of being wide in energy to excite both the lower and upper polariton modes so as to lead to Rabi oscillations of the vortex packet in the photon and exciton fields. More specifically, we consider the case where the first pulse brings the topological charges while the second pulse, tightly focused in space, brings the interference. Upon being superimposed, an instantaneous nonzero NTLM [7] is induced in each field, despite each initial pulse bringing zero net linear momentum (for symmetry reasons the NTLM is zero also in a plain vortex beam). This is due to the loss of translational symmetry in the plane caused by the displaced vortex, so that linear momentum is not conserved in the cavity and the wavepacket can acquire an NTLM in a given direction, as is further discussed below. When, after a delay, the second tight pulse arrives, it displaces the vortex cores set by the first pulse and triggers the SIP regime. This mixed dynamics creates a dislocation in the interference fringes which is spiraling with the Rabi frequency. Such a dislocation comes from the rings of the vortices being displaced by the Rabi-oscillations of their cores. The dynamics gives rise to a new time-varying feature of the OAM, that is, the OAM oscillates in sync with the Rabi oscillations, which are ultrafast and now transported in space. The loss of spatial overlap between the upper and lower polariton modes leads to the decrease of the oscillations in time, with no loss of coherence in the system. We also describe the topology of the fields from the perspective of polarization ellipses which is common in singular optics [31, 32], where we use the pseudo-spin representation of polariton states, where our beam is analogous to a Poincaré beam [33], with an associated elliptical field that varies in space and time.

The paper is organized as follows. In Section 2 we provide the theoretical model and describe the pumping schemes that result in a rotating wavepacket in the SIP regime. In Section 3, we provide the main results and in Section 4, we provide concluding remarks.

## 2 Theory

Our binary Bose system is described by two coupled Schrödinger equations [34]

(1) $$\text{i}\hbar \partial_{t}\left(\begin{array}{c} \psi_{\text{C}}\left(x,y,t\right)\\ \psi_{\text{X}}\left(x,y,t\right) \end{array}\right)=L\left(\begin{array}{c} \psi_{\text{C}}\left(x,y,t\right)\\ \psi_{\text{X}}\left(x,y,t\right) \end{array}\right),$$

where

(2) $$L=\left(\begin{array}{cc} -\frac{\hbar^{2}\nabla^{2}}{2m_{\text{C}}}+E_{\text{C}} & \hbar \Omega \\ \hbar \Omega & -\frac{\hbar^{2}\nabla^{2}}{2m_{\text{X}}}+E_{\text{X}} \end{array}\right).$$

We work in the regime of large mass imbalance (*m*_X_ ≫ *m*_C_) corresponding to exciton-polaritons in optical microcavities [16]. As such, *m*_C_ and *m*_X_ are the effective masses for the cavity photon (*ψ*_C_) and exciton (*ψ*_X_) fields, respectively. The strong (Rabi) coupling between the two fields is given by the Rabi-splitting energy *ℏ*Ω. We do not include dissipation, as our emphasis is on the interplay between the Rabi-oscillating vortices and SIP. The real system may suffer from decay and/or dephasing [20], however we put such effects aside as they mainly result in quantitative differences. Their main effect consists in a further damping of the oscillations with respect to that due to the loss of spatial overlap here discussed. We also point out that in the case of no decay and hence zero width of the polariton linewidths, in principle the system would always be in the strong coupling regime, for any finite coupling strength. However, the energy splitting here used (about 8 meV, see Figure 1(a)), is of the same order of magnitude as what is observed in real samples, as for the other parameters. Typical linewidths in high quality samples can be one order of magnitude smaller than the coupling strength, resulting in the possibility to observe tens of Rabi oscillations, as previously reported [8, 10]. To start the dynamics, we use pulses to pump the system by an external source, describing for example a laser. From the different schemes of pumping, we focus on the pulsed resonant pump of different spatial sizes directly injecting particles in the photon field only. Consequently, a new term is added to the right hand side of Eq. (1),

(3) $$\left(\begin{array}{c} \sum_{j}p_{j}\\ 0 \end{array}\right).$$

We assume Laguerre–Gaussian (LG) profiles for the pulses (in polar coordinates $r=\sqrt{x^{2}+y^{2}}\;and\;\varphi =\arg\left(x+\text{i}y\right)$ ):

(4) $$p_{j}\left(x,y,t\right)=R_{j}{\left(\frac{r}{W_{j}}\right)}^{\left|l_{j}\right|}{\text{e}}^{\text{i}l_{j}\varphi }{\text{e}}^{-r^{2}/2W_{j}^{2}}{\text{e}}^{-{\left(t-t_{j}\right)}^{2}/2\delta_{t}^{2}}{\text{e}}^{\text{i}\omega_{j}t}$$

where *l*_*i*_ is the winding number of the field and *R*_*i*_ is the pumping amplitude. Here, *W*_*j*_ is the parameter to control the wavepacket size; the pulse is being sent at the time *t*_*j*_ with frequency *ω*_*j*_, and its duration is given by *δ*_*t*_. Initially, we assume *ψ*_C_(*x*, *y*, 0) = *ψ*_X_(*x*, *y*, 0) = 0, and the system is pumped through the sequence of pulses as previously described. Namely, we solve Eq. (1) numerically and consider its time dynamics where we first pump the system with a *l*_1_ = 1 pulse and after a delay, send the second pulse with *l*_2_ = 0. Here, the spot size of the first pulse *W*_1_ is chosen not to go beyond the inflection point (extended vortex packet), that is, the polariton dispersion is parabolic. For the second pulse, the (spatial) size of the pulse spot *W*_2_ is chosen to be so tight as to excite the polaritons also beyond the inflection point, so the dispersion is not parabolic, as is further discussed in the Supplementary Material. In absence of Rabi oscillations, the use of different LG pulses is known to underly the shaping of off-axis vortices [7], full Bloch beams with spiraling vortices [8] and even 3D skyrmionic textures [35].

## 3 Results

 In the following, we first consider the real-space dynamics; then we study the dynamics of the quantum states, i.e., as mapped to the Poincaré sphere (meant here as the pseudo-spin space describing the binary polariton state, alternatively called the Bloch sphere of states), thanks to which we can perform a polarimetric analysis to identify the so called C-points. Finally we discuss the time-varying feature of OAM.

### 3.1 Dislocation propagation

Based on the pumping scheme previously introduced, we first pump the system with the pulse *p*_1_, where a topological charge is brought first to the photon field and then, due to the Rabi oscillations, the field is transferred along with the topological charge to the exciton field. As the transfer proceeds, the topological charge gets distributed in both fields simultaneously. Equivalently, there are two cores in the polariton fields, but they are now, like the fields themselves, stationary. The density map of any of these fields includes a central null point (vortex core), where the density is zero. After performing some Rabi oscillations, the second pulse is introduced, to occupy a small region in real space, therefore with a fast diffusion. Also, the action of the second pulse is to immediately displace the vortex cores because of the incoming pulse interference with the previously created polaritons. As long as the wavepacket expands radially in space without interferences, its contribution to OAM can be well identified. The interference of the radial and azimuthal flows sculpts out spiral waveforms whose contributions to OAM can no longer be tracked. The Rabi oscillations and the SIP effect result in an interesting new phenomenology.

**Figure 2:**
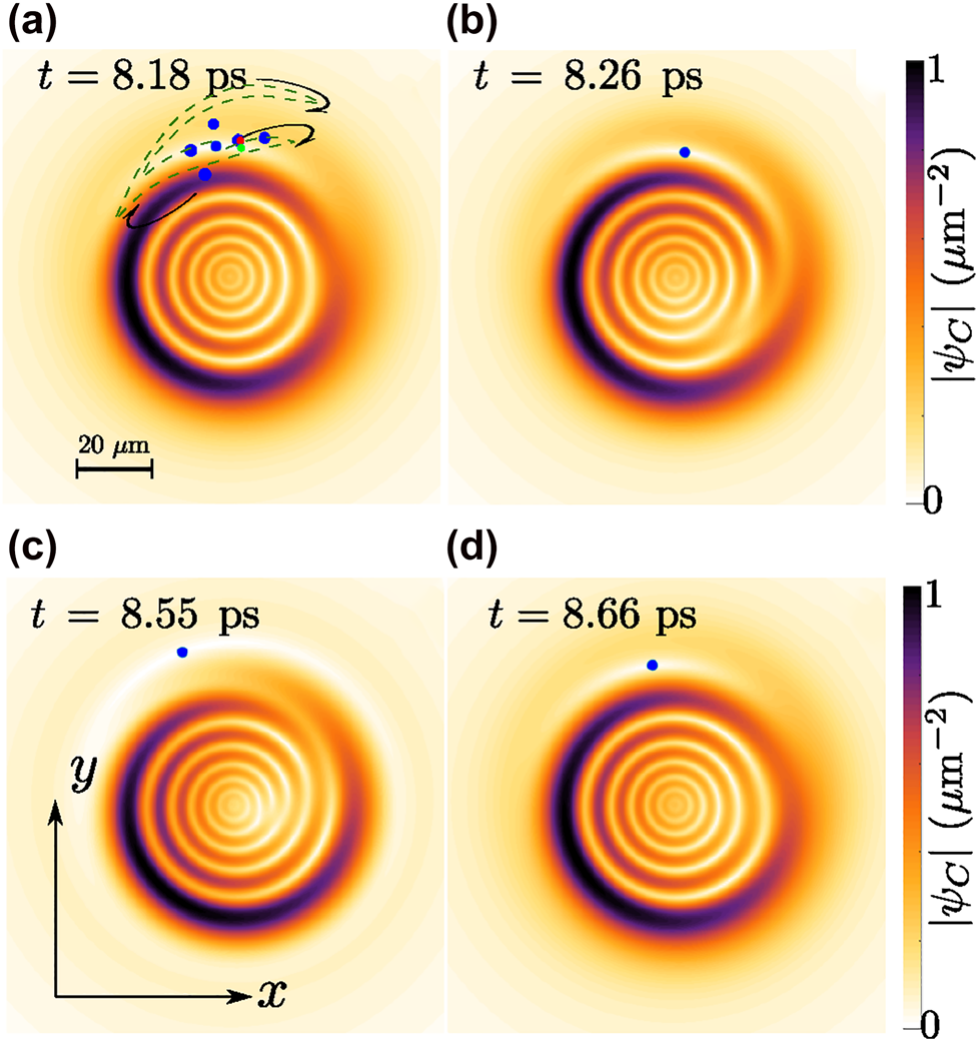
The regime of Rabi interference dislocation, shown in the amplitude of the photon component of polaritons. After sending the second pulse, the mixed dynamics of SIP and the moving Rabi vortices produce a moving dislocation in the pattern. The dislocation stems from the misalignment of the rings, induced by the motion of the vortex core. Here, different frames along one Rabi period have been chosen. In each frame, the position of the vortex core is shown by a blue point. It follows an expanding orbit that is shown (in one period) by the dark-dashed green line in the first panel. The starting point is marked as a green dot and the end point as a red one. For the numerical simulations, we used: *ℏ*Ω = 4 meV, *ω* = −Ω/3, *W*_1_ = 5 μm, *W*_2_ = 1.2 μm, *t*_1_ = 0, *t*_2_ = 1.5 ps, *δ*_*t*_ = 0.3 ps, *R*_1_ = 1 ps^−1^, and *R*_2_ = 4 ps^−1^.

We present in Figure 2 the amplitude map of the photon field. Among different fields inside the cavity, only the photonic part, that escapes the cavity, can be detected via external devices. By using a spectral resolution or filtering tool, the upper and lower modes could be also detected separately in their photonic component, at the price of loosing some time resolution though. As we expect from the physics of SIP, rings of high and low densities are created; but there are new features due to the topology of the initial vortex. First, the rings of high (or low) densities are not symmetric (high density is, in this case, shifted to the left) whereas they remain symmetric in the absence of the vortex (see panel (c) of Figure 1). This is similar to the physics of the displaced vortices [9], but here this happens for the rings. The displacement can therefore occur in any direction, depending on the relative phase. This could have applications for clocking, e.g., by transforming a time delay into an angle. A symmetric wavepacket, such as a Gaussian, has a mean linear momentum ⟨−i*ℏ*∇⟩ equal to zero. There is a diffusion of the wavepacket but this never leads to an overall or average motion, that is, the centroid of the wavepacket does not move. This changes for an asymmetric wavepacket, such as a displaced vortex state in the SIP regime, which can acquire an NTLM from the asymmetry in the system without any linear momentum being brought from the outside. To describe this, we compute the linear momentum [36]

(5) ⟨pi⟩=ℏ∫dxdyImI^ψi*∂xψi+J^ψi*∂yψi.

**Figure 3:**
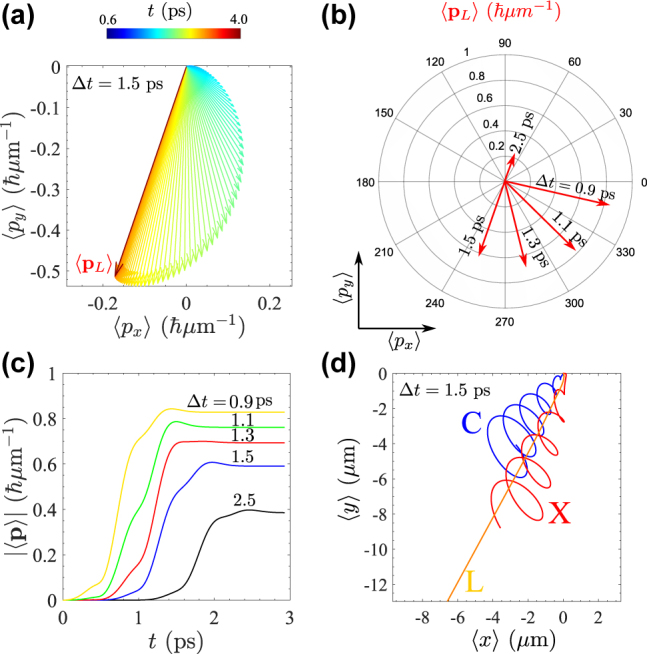
(a) Creation, evolution and stabilization of the NTLM vector, here for the lower polariton field. The final direction is shown by the red vector. (b) Different values of Δ*t* (time delay between the pulses) yield different final NTLM directions. (c) Amplitude of the total linear momentum ⟨**p**⟩ = ⟨**p**_C_⟩ + ⟨**p**_X_⟩ = ⟨**p**_L_⟩ + ⟨**p**_U_⟩ for different Δ*t*. Linear momentum is created with the second pulse and remains constant when its direction stabilizes. (d) Wavepacket centroids for various fields, showing with curly curly oscillations of the bare fields and straight linear motion for the dressed ones (the small deviations in the L direction at earlier times are not visible on this scale).

Here, ⟨**p**_*i*_⟩ is the NTLM of the $i=\left\{\text{C,X,L,U}\right\}$ fields. Typical variations of ⟨**p**_*i*_⟩ for the lower polariton wavepacket are shown in Figure 3(a). Since the upper packet diffuses fast, its associated NTLM is almost negligible and hence we are focused on the portion in the lower packet. The arrows show the evolution of the NTLM’s direction in time (with a time step between each arrows of 0.01 ps, the magnitudes are shown in panel (c)). Upon sending the second pulse, the transverse linear momentum (TLM) is formed and its net value eventually points in a fixed and stationary direction in the dressed (upper and lower polariton) fields, while it oscillates in the bare (photon and exciton) fields. Interestingly, the fixed direction of the NTLM can be manipulated by the time delay between the two pulses. In panel (a), we assume a time delay of Δ*t* = 1.5 ps and in panel (b), different values of Δ*t* result in a different final direction. This allows to trigger and orientate a net motion of the centroid of the wavepacket (⟨r⟩=I^⟨x⟩+J^⟨y⟩) in the SIP regime. Panel (d) shows the trajectories of ⟨**r**⟩ for the bare and the lower polariton wavepackets, contrasting the spiral trajectories of the former as opposed to the fixed direction maintained by the NTLM for the polariton. Indeed, by tuning the time delay, a propagating wavepacket can be oriented in any specific direction. This may have applications in atoms/molecules segregation [37] or phase transformation [38].

Other interesting features pertain to the time dynamics. For instance, rather than a mere diffusion of the ripples, they oscillate with the same rhythm as the vortex core motion, which is itself induced by the Rabi oscillations. Another example follows from the azimuthal misalignment of the rings, mostly visible in the low density region. This produces a dislocation (fork-like signature) in the pattern of fringes. This is particularly visible in panels (b) and (c) of Figure 2. In panels (a) and (d), the dislocation is also there but repelled at the frontier of the packet, where it is less visible. This is due to the motion of the vortex core positioned in the outermost region, adding an extra low density ring to the inner ripples. The dislocation oscillates at the Rabi frequency, since the vortex core does too. Dislocations or forks generally appear in interference patterns and are associated to vortices. For example, interference of a vortex with a plane wave or a vortex with itself can result in the emergence of a dislocation [39]. However, the appearance of a dislocation in the SIP regime is of a different origin, namely, the displaced vortex changes the alignment of the ripples, inducing a dislocation at distances far from the vortex itself. We provide movie animations of each density profile in the Supplementary Movies SM1-SM4 (see Supplementary Material for descriptions). An additional movie SM5 is also attached that displays the dynamics of a slice of the photon density near the maximum point of the density (contour of Max|*ψ*_C_|(1 − *ϵ*), where *ϵ* is small). This shows clearly a see-saw motion for the NTLM, rhythmed by the Rabi oscillations. This could have application for a more forceful, sweeping particle clearing [40].

**SM1:**

**SM2:**

**SM3:**

**SM4:**

**SM5:** 

### 3.2 Polarimetric analysis: topology imaging

The concept of transverse light polarization naturally arises in many optical situations and its spatial distribution or texture can be non-homogeneous. The phase singularity in a plain quantum vortex can become a polarization singularity in so-called spin vortices. For a scalar field, topological defects of two kinds can be found by looking at the phase isolines orientation [41, 42] and their textures, with a negative integer winding (−1) for saddle points and positive (+1) for dislocations. Dislocations, also known as phase singularities or phase vortices, can be further distinguished by their other quantum number (−1 or +1), associated to the phase winding around the singularity. In the spin vortices of a vector field, the textures can be associated to the polarization of the fields, e.g., also encoded in the orientation and eccentricity of the polarization ellipses [43–47]. In such a case, and similar to polarization states in optics, we can extend the representation and concepts of the ellipse geometry to our binary field (see Supplementary Material for further details). Namely, we use a Poincaré or Bloch sphere representation, associated to the complex polariton state, and choose the dressed modes (upper and lower) basis as the vertical axis of such a sphere. One can define the quantity

(6) $$\sigma \equiv \psi_{U}^{*}\psi_{\text{L}},$$

where the nodes of *σ* give the circular state (or C-points [31, 32]) of the elliptical field, and also the vortex core positions. Indeed, here the C-points are representing a pure lower (upper) state; and as such they require a zero density in the opposite upper (lower) field. For such a reason, a vortex in one field is also a C-point in this representation (the vortex core bears a zero density so it is a pure state of the opposite field). Also, the phase of the quantity in Eq. (6) is the relative phase between the upper and lower fields, arg[*σ*] = arg[*ψ*_L_] − arg[*ψ*_U_].

**Figure 4:**
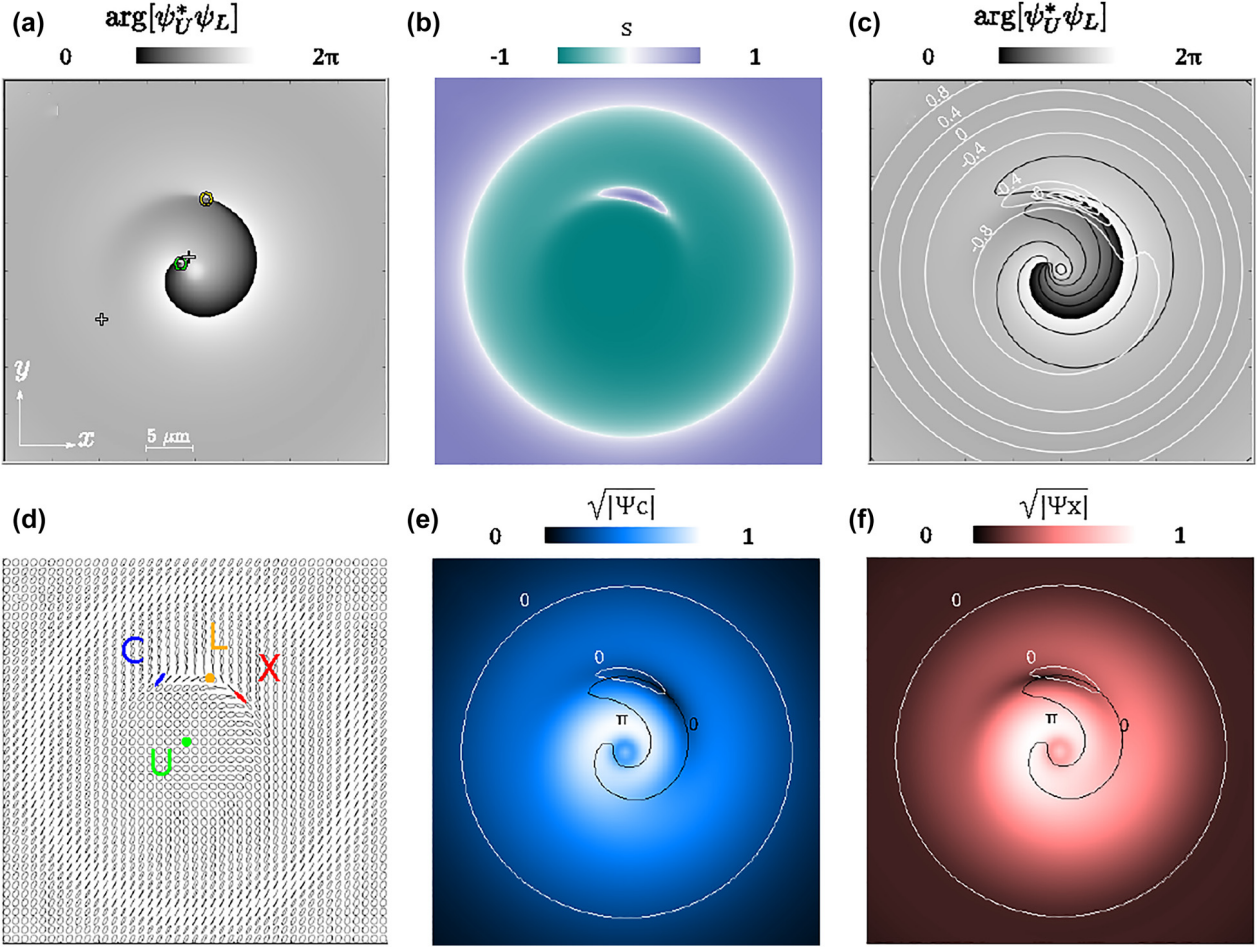
Various representations of the fields morphology, namely, based on their relative phase $\sigma =\arg\left[\psi_{\text{U}}^{*}\psi_{\text{L}}\right]$ and amplitude $s=\frac{\left\{|\psi_{\text{U}}{|}^{2}-|\psi_{\text{L}}{|}^{2}\right\}}{\left\{|\psi_{\text{U}}{|}^{2}+|\psi_{\text{L}}{|}^{2}\right\}}$ (in upper–lower polariton basis), isolines of *σ* and *s*, ellipses (in upper–lower polariton basis), and amplitude (the square root is used to enhance the contrast) of the photon and exciton fields together with vortex cores and saddle points, at *t* = 3.15 ps. (a) The phase map of *σ*, also shows the positions of vortices in the upper–lower basis. Indeed, the unitary vortex charges in each of the two fields compose a vortex dipole in the relative phase map. The displaced vortices (located at yellow and green rings) also induce two saddle points (indicated by two cross symbols) in the relative phase. (b) The relative amplitude *s* (or equivalently the amplitude of quantum states) varies between −1 and 1 and has an inhomogeneous profile. Panels (a) and (b) imply that all quantum states of the Poincaré sphere are simultaneously present in the 2D space. (c) The isolines of relative phase (shown as black curves, at *π*/3 intervals) are superimposed with isolines of the relative amplitudes: s (white lines, at 0.4 intervals). (d) Elliptical representation of the polariton state and its spatial variations in 2D space. The green (yellow) point, where the state of the ellipse becomes a circle, corresponds to the upper (lower) vortex core. The state of the ellipse is linear at the exciton and photon vortex cores (blue antidiagonal and red diagonal, respectively). Indeed, at the exciton and photon vortex cores, the polariton state is purely photonic and excitonic, respectively. (e) Squared amplitude of the photon field, with the position of the vortex core at intersection of *s* = 0 and *σ* = 0. (f) Squared amplitude map of the exciton field, with the position of the vortex core at the intersection of *s* = 0 and *σ* = *π*. The vortex cores in real space move along the inner white curve during a Rabi cycle.

An example of the structuring of the complex function *σ* is given in Figure 4, where the panels show the morphology based on the relative phase and amplitude map—phase and amplitude isolines—and ellipses representation, respectively, together with the C-points. Panel (a) shows the positions of the singularities, or vortex cores, overlapped to the phase map (arg[*σ*]). The vortex core in the upper field (green point) is connected to the vortex core in the lower field (yellow point) via relative phase isolines of all possible values (whereas the 2*π* isoline is the one clearly visible in the map). A space chart of $s=\frac{|\psi_{\text{U}}{|}^{2}-|\psi_{\text{L}}{|}^{2}}{|\psi_{\text{U}}{|}^{2}+|\psi_{\text{L}}{|}^{2}}$ is shown in panel (b). This effectively defines the latitude of a quantum state on the Bloch sphere (see the Supplementary Material for further details). Interestingly, *s* is not homogeneous in space. While the relative phase maps also the longitude of the Bloch sphere, the value of *s* that varies in the interval [−1, 1], maps the latitude on the sphere, with *s* = cos(*θ*) with *θ* the polar angle. The parameter *s* is +1(−1) at the vortex core of the lower (upper) polariton, while it is 0 at the positions of equal upper/lower content. The C, X vortex cores are moving in an orbit described by the *s* = 0 line. Correspondingly, the isolines of relative phase along with the isocontent *s*, are shown in panel (c) (black and solid lines, respectively). The panel (d) shows the corresponding elliptical representation. The ellipse is a very useful tool to describe the polarization state of the electric fields in optics, as well as any other vector field, both in 3D as well as in 2D space. In particular, its eccentricity and orientation can be associated to the (latitude and longitude of the) points on the surface of a unique topological structure, namely the Poincaré sphere. Similarly here, the spatial distribution of normalized ellipses, describing now the binary polariton state on a Bloch pseudospin sphere, is shown in panel (d). Here we find two kinds of C-points; one indicated by a yellow circle, that corresponds to the lower state, and another depicted by a green circle, associated to the upper vortex core. They are associated to opposite winding of the relative phase around each singularity. At the C-points, the ellipses become circles, and one can find the opposite winding of the ellipses axis around two different C-points. Indeed, the orientation of the ellipse axis is directly mapping (half of) the longitude angle on the sphere, which is given by the relative phase. The typical texture of a so-called star spin vortex is visible around the lower mode vortex core (yellow C-point), while the opposite so-called lemon texture is more spread in space around the upper mode core (green C-point), and hence less recognizable. The two X, C vortex cores are associated to a diagonal, antidiagonal linear state (red and blue segments in the panel). They move, thanks to the Rabi oscillations, along the inner closed orbit, around the lower mode vortex core (Figure 4(e) and (f)). Such an orbit is also the *s* = 0 isocontent line, and represents a so called L-line (the one where the star pattern is visible). The external L-line, the big circle in panel (c), does not represent an orbit, in the sense that it has the same linear elliptical state at all times. The orientation of the ellipses are varying in space, describing some 2D domain on the sphere [33]. The texture of the sphere coordinates (longitude and latitude), represented by arg[*σ*] and *s* isolines, is not conformally mapped to the real space (the two isoline families are not mutually orthogonal everywhere in space, as they are on the sphere) differently from the conformal full Bloch beams [8, 44]. We ascribe this to the achieved configuration being a superposition of LG_00_ and LG_01_, both with the same center but not the same width. However, the integral of the Bloch sphere surface density in real space is equal to 4*π*, hence suggesting that the emitted beam is analogous to a distorted full Poincaré beam, where sphere texture is not conformal to the real space.

### 3.3 Orbital angular momentum oscillations

In a standard symmetric vortex, the amount of rotation of the density is not directly visible, since for symmetry reason, the intensity ring remains despite of the rotation in the phase, that is nevertheless able to exert a physical torque. Indeed, the symmetric vortex has a total OAM ⟨*L*_*z*_⟩ and an OAM per particle $\left\langle {\tilde{L}}_{z}\right\rangle \equiv \left\langle L_{z}\right\rangle /N$ , where we define [36]

(7) $$\left\langle L_{z}\right\rangle =-i\hbar \int \psi^{*}\partial_{\varphi }\psi rdrd\varphi ,$$

(8) $$N=\int \psi^{*}\psi rdrd\varphi .$$

In the standard symmetric vortex of unitary charge, the OAM per particle is unitary as well, while if the core is off-axis, also leading to asymmetric shapes, the OAM per particle can be fractional [7, 48, 49]. It is therefore interesting to find what is the OAM per particle in our structured case, that not only displays offset vortex cores but also spiral patterns, that are furthermore reshaping in time.

**Figure 5:**
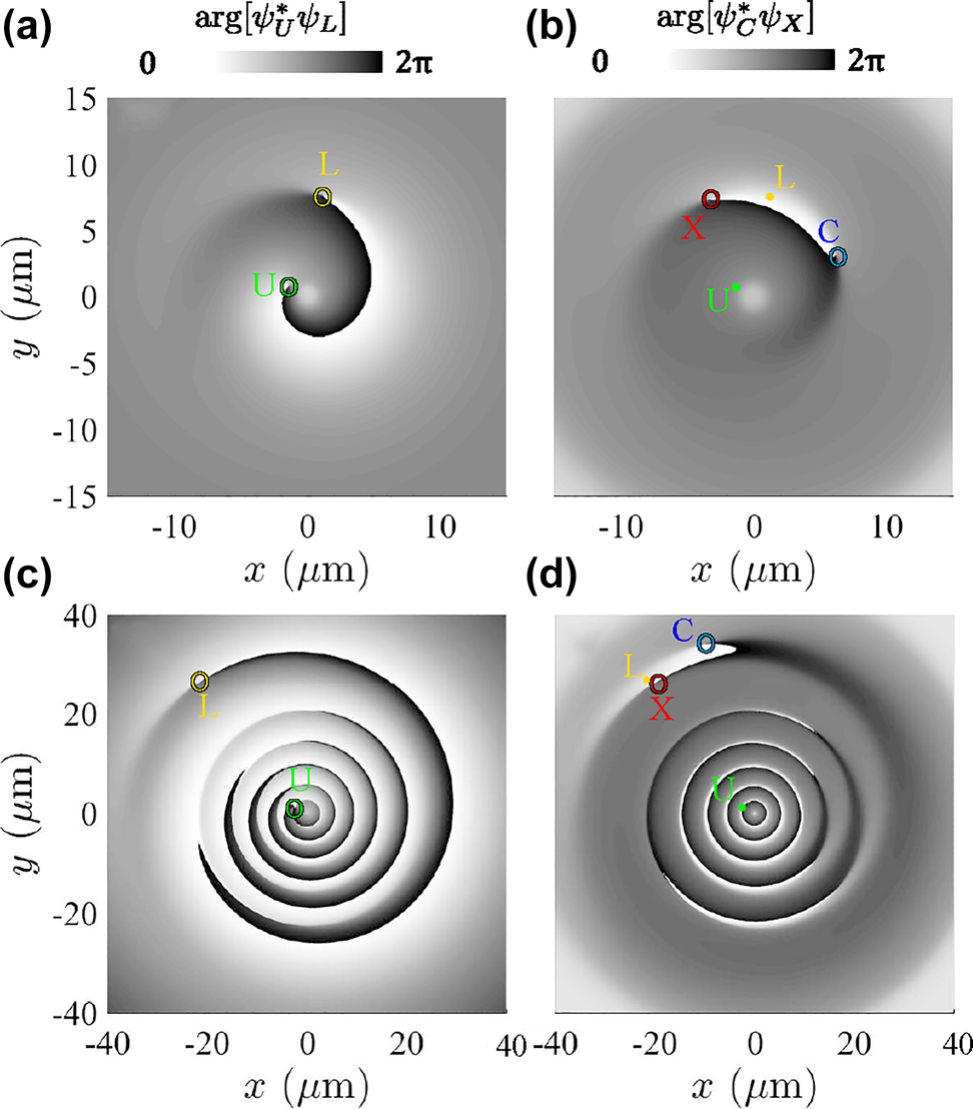
Relative phases and vortex cores positions at different times. (a) and (c) Show the phase map of $\sigma =\psi_{\text{U}}^{*}\psi_{\text{L}}$ , which is the relative phase between the upper and lower fields, at *t* = 3.15 ps and *t* = 8.14 ps, respectively. The corresponding phase map of $\psi_{\text{C}}^{*}\psi_{\text{X}}$ , between the exciton and photon fields, is shown in panels (b) and (d), at the same times as (a–c). The position of the vortex cores in all the fields are shown by C, X, L, U labels (for photon, exciton, lower polariton and upper polariton). The upper mode, excited by the second and tight pulse, undergoes a rapid diffusion, and the initial vortex core remains little affected by interferences, remaining almost at the center of the packet, close to the origin of the 2D map (green circle). The core in the lower mode experiences a larger interference with the tight excitation diffusing more slowly, for such a reason it is displaced at the boundary of the packet (yellow circle). The core in the lower mode is then set aside due to the delayed arrival of different radial momenta (changing the interfering phase and hence azimuthal position of the vortex core). However, the cores in the photon and exciton fields are displaced at the boundary as well, and due to Rabi oscillations, they orbit, e.g., around the lower mode core. Differential diffusion between the two normal modes then leads to a loss of their overlap, damping the Rabi oscillations and reducing the C, X vortex cores orbits.

To answer this, we consider the phases involved in the dynamics at two given times, close to the arrival time of the second pulse and after a few picoseconds, as shown in Figure 5. Each row (Figure 5(a)–(d)) corresponds to a time, while each column corresponds to the relative phase between the various fields involved (Figure 5(a)–(d) for the photon–exciton and lower–upper relative phase, respectively). Shortly after sending the second pulse, the vortex cores in each fields are displaced. The simplest dynamics happens for the core in the upper field. Due to a fast diffusion of the upper polaritons injected by the second pulse (which is very tight), the vortex core is less affected by interferences and is displaced only slightly from the origin, where it remains almost at all times (green circle or label in Figure 5). Such a feature of the dynamics can be partially controlled by the energy of the pulse (*ω*), namely, for more negative energy, the core in the upper field is less displaced.

The dynamics for the other core is different. Indeed, the fraction injected by the second pulse, despite being a tight beam, is subject to the group velocity limitations of the lower polariton mode, and undergoes the SIP effect. Only a portion of the momenta composing the packet travels fast outwards. The remaining part, filling the center, moves the vortex core of zero density at a larger distance, as a result of interferences (yellow circle or label in Figure 5). The combination of structured diffusion and azimuthal phase winding from the vortex, results then in the greater lateral movement of the core in the lower field (panels a, c). Hence, it can be seen that the OAM per particle has been altered in both of the normal modes, due to the superposition of the two pulses, and this is reflected in their vortex cores displacements. However, the photon and exciton fields oscillate due to the Rabi oscillations, and their vortex cores orbit along some path (blue and red circles or labels in Figure 5), which is reflected in their OAM oscillations. As far as the differential diffusion between the normal modes reduces their overlap, both the OAM oscillations decrease in time as the orbits shrink.

**Figure 6:**
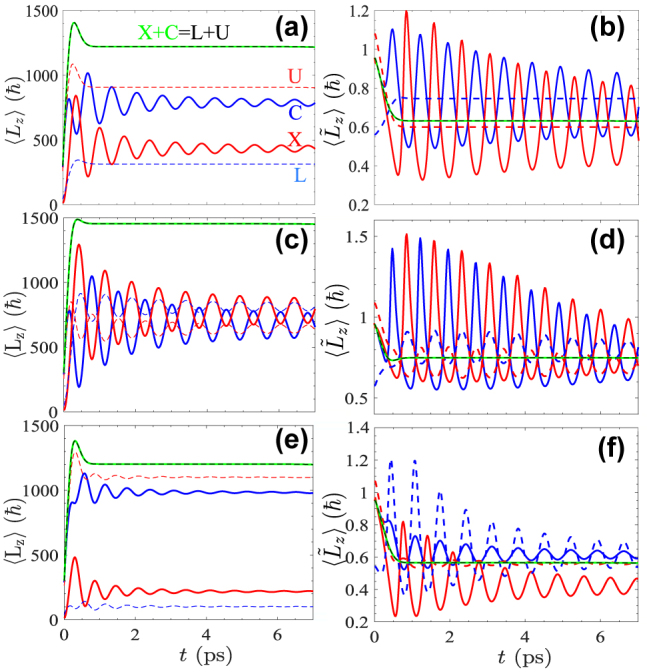
Time-varying orbital angular momentum. Although there is no decay of the fields, oscillations dampen in time. This is due to the fast diffusion of the packet in the upper mode and the consequent loss of overlap with the lower mode. This happens in a small fraction of the polariton lifetime and can be engineered either by energy or packet size manipulations. In (a–b) the energy detuning is *δ* = 0. We assume *δ* = 1.5 in (c–d) and *δ* = −1.5 in (e–f). We used *W* = 2 μm for the first pulse and *w* = 0.5 μm for the second pulse.

Such a structured packet can be engineered by tuning the time delay between the two pulses and/or the size of the wavepackets. The key point is that for smaller wavepackets, the Rabi oscillations go off faster. After the second pulse is received and the vortex core is displaced, the wavepacket undergoes density rotation. As long as the Rabi oscillations continue in time, the wavepacket carries extrinsic OAM and rotates. We show in Figure 6 the time-varying total OAM and OAM per particle for different cases. A common feature of these panels is that the oscillations attenuate in time. The rate at which the oscillations decrease depends on the wavepacket size, laser energy detuning, and the energy detuning between the bare fields. Their combination eventually accounts for the reduction of the overlap of the injected *l* = 0 fraction with the previous *l* = 1 part, faster in time when using smaller packets (which is, for tighter beams) and for more negative laser energy. Here, the oscillations in $\left\langle {\tilde{L}}_{z}\right\rangle$ implies a varying distance of the vortex core in a given field. Compared to the previously discussed time-varying OAM [8], [9], [10], here the oscillations stop in a few picoseconds, Figure 6(a) and (b), despite the modes having no decay and no central linear momentum (i.e., no NTLM) being imparted. This comes fully from the differential diffusion of the packets, which separates the dressed fields in a few picoseconds. We also consider the effect of energy detuning, defined as *δ* = *E*_C_ − *E*_X_. A positive *δ* can trigger more oscillations, and also larger OAM per particle $\left\langle {\tilde{L}}_{z}\right\rangle$ as shown in Figure 6(c) and (d). For a negative detuning, the oscillations are fading away very fast, as shown in Figure 6(e) and (f).

## 4 Conclusions

A quantum vortex, i.e., a phase singularity in a quantum fluid, is usually associated to the OAM per particle, whose value depends on the shape of the surrounding fluid. It may be associated to a dislocation of the density too, a so-called fork-like shape. In this paper, we identified a new type of moving dislocation obtained from the interference of radial flows of packets sharply localised in real space with azimuthal flows due to the presence of a vortex. This scenario can be realized with a two-pulse scheme that sets the initial condition, while the subsequent dynamics is ruled by the mass differences in a binary field, leading to the creation of a so-called self-interfering packet. This structuring happens in the linear regime by combining the peculiar lower polariton dispersion with vorticity.

The specific pattern of the spatially varying relative phase between two normal modes of different eigen-frequencies, is the underlying mechanism behind the self-shaping of the rotating interfering patterns with a fork-like feature. The experimental implementation of these dynamics involves two Gaussian pulses sharp enough in time to excite both polariton modes simultaneously at normal incidence (thus with zero linear momentum), with the first beam passing through a *q*-plate to imprint the vortex and the second beam being tightly focused in space to diffuse fast and extend over a large span of momenta, beyond the inflection point of the dispersion relation. The moving dislocation is associated to a time-oscillating OAM per particle, which gets damped due to the reducing overlap of the polariton modes in time. This is due to the different speeds at which the two (normal) fields diffuse. This results in time and space structuring of a packet and oscillations of its OAM. Beyond the fundamental interest of such topological dynamics created by the interplay of strong coupling with vorticity, the see-saw motion of in-plane oscillations could lead to applications if the field can be coupled to microscopic objects.

## Supplementary Material

Supplementary Material Details
