# Supplementary material for: Topologically driven Rabi-oscillating interference dislocation
Source: Nanophotonics. 2022 May 9;11(12):2909–19. doi: 10.1515/nanoph-2022-0108 (PMC11501282; doi:10.1515/nanoph-2022-0108)
Supplement: Supplementary file 11 — Supplementary Material Details [file j_nanoph-2022-0108_suppl.pdf]

Topologically driven Rabi-oscillating interference dislocation

Supplementary Material

I. POLARITON DISPERSION

In this appendix section we describe the peculiar dispersion of the polaritons. To this end, we need to switch to momentum space through Fourier transform $\mathcal{F}$; in this space we use tilde symbol for representing fields; then, $\tilde{\psi}_{C,X} = \mathcal{F}[\psi_{C,X}]$. Starting with the matrix representation in Eq. (1) in the main text, one can find its counterpart in reciprocal space as:

$$\tilde{\mathcal{L}} = \begin{pmatrix} \frac{\hbar^2 k^2}{2m_C} + E_C & \hbar\Omega \\ \hbar\Omega & \frac{\hbar^2 k^2}{2m_X} + E_X \end{pmatrix} . \tag{1}$$

If we define the lower and upper polariton states in momentum space through respectively $\tilde{\psi}_L = -C_X\tilde{\psi}_X + C_C\tilde{\psi}_C$ and $\tilde{\psi}_U = C_C\tilde{\psi}_X + C_X\tilde{\psi}_C$, then the matrix

$$\tilde{\mathcal{M}} = \begin{pmatrix} C_C & -C_X \\ C_X & C_C \end{pmatrix} . \tag{2}$$

diagonalizes $\tilde{\mathcal{L}}$ through similarity transform $\tilde{\mathcal{M}}\tilde{\mathcal{L}}\tilde{\mathcal{M}}^{-1}$. Thus we can find an expressions for coefficients C_C

$$C_X = \frac{1}{\sqrt{2}}\sqrt{1 + \frac{\Delta E}{\sqrt{\Delta^2 + 4(\hbar\Omega)^2}}} , \tag{3}$$

and also for $C_C = \sqrt{1 - C_X^2}$. Here we introduce $\Delta \equiv \frac{\hbar^2 k^2}{2m_C} - \frac{\hbar^2 k^2}{2m_X} + \delta$, and $\delta = E_C - E_X$. Finally we have the following results for the lower and upper polartion dispersions, respectively, as:

$$E_{L,U} = \frac{1}{2}\Big[\frac{\hbar^2 k^2}{2m_C} + \frac{\hbar^2 k^2}{2m_X} \pm \sqrt{4(\hbar\Omega)^2 + \Delta^2}\Big] , \tag{4}$$

Particular example of the dispersion is shown in Figure 1a for $\delta = 0$. Analytical solutions can be found for fields in Fourier space. We define two dressed states of lower and upper polaritons:

$$\begin{pmatrix} \tilde{\psi}_L \\ \tilde{\psi}_U \end{pmatrix} = \mathcal{M} \begin{pmatrix} \tilde{\psi}_C \\ \tilde{\psi}_X \end{pmatrix} = \begin{pmatrix} C_C & -C_X \\ C_X & C_C \end{pmatrix} \begin{pmatrix} \tilde{\psi}_C \\ \tilde{\psi}_X \end{pmatrix} , \tag{5}$$

where $C_{C,X}$ are Hopefiled confinements. These dressed states admit the following equations of motion:

$$i\hbar\partial_t \begin{pmatrix} \tilde{\psi}_L \\ \tilde{\psi}_U \end{pmatrix} = \mathcal{J} \begin{pmatrix} \tilde{\psi}_L \\ \tilde{\psi}_U \end{pmatrix} + \mathcal{P}(t) , \tag{6}$$

where we define $\mathcal{J} \equiv \mathcal{M}\mathcal{F}[\mathcal{L}]\mathcal{M}^{-1}$, which is a diagonal matrix, $\mathcal{P} \equiv \begin{pmatrix} C_C\mathcal{F}[\sum_{j=0} p_j(t)] \\ C_X\mathcal{F}[\sum_{j=0} p_j(t)] \end{pmatrix}$, and $\mathcal{F}$ stands for Fourier transform. We can find the solutions as ($\tilde{\psi}_{U,L}(t = 0) = 0$):

$$\tilde{\psi}_L = -\frac{iC_C}{\hbar} e^{-\frac{iE_L t}{\hbar}} \int_0^t e^{\frac{iE_L t'}{\hbar}} \mathcal{F} \sum p_j(t') , \tag{7a}$$

$$\tilde{\psi}_U = -\frac{iC_X}{\hbar} e^{-\frac{iE_U t}{\hbar}} \int_0^t e^{\frac{iE_U t'}{\hbar}} \mathcal{F} \sum p_j(t') , \tag{7b}$$

where $E_{L,U}$ are the dispersion for lower and upper polariton branches.

II. SELF-INTERFERING WAVEPACKET

We explained in the main text that the lower polariton dispersion is non-parabolic beyond its inflection point k_{ip}. Considering group velocity of the lower polariton, it obtains a local maximum at this inflection point. If the wavepacket's spread goes beyond such a point, self-interfering patterns appear in the dynamics of the wavepacket. The physics behind this regime goes like this: the states of high and low momenta propagate together at slower speed, with respect to state associated to the inflection point momentum which is faster. As a consequence, the content of momenta gets separated and is not continuous anymore, and the two "discretized" momenta that remains overlapped, result in interference patterns or ripples, i.e, symmetric regions of low and high densities. Example of such ripples is shown in panel (a) of Figure S1 and of Figure 1 in the main text. It is worth noting that the upper dispersion has always a positive curvature and its associated wavepacket propagates in the same way as a free particle, and consequently we do not expect the ripples in the upper field. We also note that such interesting physics occurs either with sharp wavepackets in real space or a wide wavepacket carrying a center linear momentum (an example of last case if presented in Figure S1, panel (b)). In this paper we deal with the former case, that is, no linear momentum is imparted and wavepackets are tight in real space.

So far we have discussed how SIP emerges in the lower polariton field, but what does we find in the photonic or excitonic components? This is important because in most of the experiments, people excite the photon field, for example by shining a laser on cavity, rather than exciting the lower branch directly (it is possible to excite eigenstates by using pulses that are spectrally narrowed [1, 2]). Again we need a sharp wavepacket in the photon component. Here alongside the SIP, Rabi dynamics also forms. The combinations of SIP in space and Rabi oscillations in time lead to the appearance of hexagonal lattices in the spacetime domain. Particular example is shown in Figure S1, in panel (d). Such a pattern can arise from the interference of three beams [3]. In our case the hexagonal lattice arises from the interference of three packets: one lower polariton at small momenta, one lower polariton at larger momenta but propagating together with the former, and one upper polariton.

III. POLARIZATION REPRESENTATIONS OF RABI-MOVING VORTICES

As a simple model to realize a fundamental case of Rabi-moving vortices we consider eigenstate solutions ψ_L and ψ_U that are approximately matched to experiment [4] and vary in both space and time [5]:

$$\psi_{L,U}(x,y,t) = \frac{A_{L,U}}{W} e^{i\frac{E_{L,U}}{\hbar}t} e^{-(x^2+y^2)/2W^2} (x - x_{U,L} + i(y - y_{U,L})) \,, \tag{8}$$

where $A_{U,L}$ stand for associated amplitudes to the upper and lower field and $(x_{U,L}, y_{U,L})$ are the vortex core positions in each field. In the photon-exciton basis corresponding fields can be found as $\psi_{C,X} = \psi_L \pm \psi_U$. We use such solutions to understand the meaning of maps, lines and ellipses in Figure 3. To do so, we recall the familiar

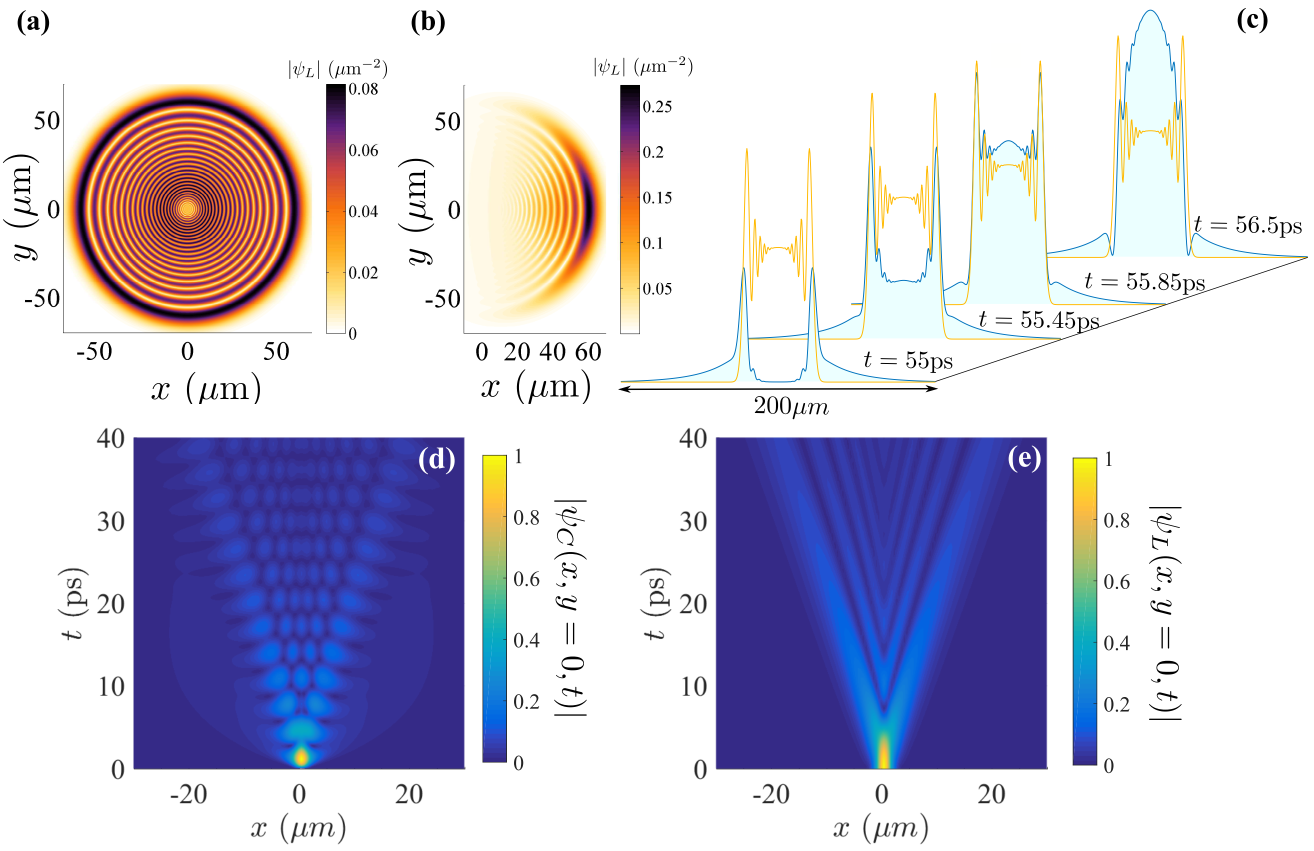

Figure S1. (a) and (b) represent the SIP in the lower polariton field, with ripples of high and low densities. In (b) there is a finite linear (added to the radial one) momentum along the x direction. (c): Cuts of interfering patterns of the photon field (shown in blue) and of the lower polariton mode (shown in orange) along the x axis. Due to Rabi oscillations, the blue patterns changes in time, but the orange patterns are stationary in time. (d-e) Spacetime patterns in the SIP regime. In (d), a spacetime hexagonal lattice is formed due to the interference of three packets: one from the upper polariton and two lower polariton packets. Corresponding patterns in the lower polariton field shown in (e). Once again we detect the regions of low and high densities, similar to (a) and (c).

concept of polarization in optics. Given an electric field in 2D space $\mathbf{E} = (E_x, E_y)$, the state of polarization is determined by the locus traced out by the real part of $\mathbf{E}$. Such a locus is in general an ellipse whose shape and orientation determine the state of polarization [6]. Alternatively, such a geometry is easily understood in terms of Stokes vectors[6]:

$$S_0 = |E_x|^2 + |E_y|^2 \,, \tag{9a}$$

$$S_1 = \mathrm{Re}[E_x^* E_y] \,, \tag{9b}$$

$$S_2 = \mathrm{Im}[E_x^* E_y] \,, \tag{9c}$$

$$S_3 = |E_x|^2 - |E_y|^2 \,, \tag{9d}$$

where the Cartesian coordinate (S_1, S_2, S_3) is a point on the Poincaré sphere. Considering a unit sphere, the associated point in the polar coordinate is given by the polar and azimuthal angles (θ_p,φ_p) defined as $\theta_p = \arg[S_1 +$

$iS_2] = \arg[E_x^* E_y]$ and $\cos\varphi_p = S_3/S_0$. Similarly, the binary nature of exciton-polaritons can be used to represent the associated states in terms of polarization ellipses or alternatively in terms of points on Poincaré sphere, i.e., $(\theta_p, \cos\varphi_p)$. The idea is to replace E_x (E_y) with ψ_U (ψ_L), and hence, a point can be defined on an analogue Poincaré sphere by introducing corresponding $(\theta_p, \cos\varphi_p) \to (\sigma, s)$ where we have $\sigma = \arg[\psi_U^* \psi_L]$ and $s = \frac{|\psi_U|^2 - |\psi_L|^2}{|\psi_U|^2 + |\psi_L|^2}$.

Given this analogy, now we consider panels of Figure S2. We deal with fields contained points where the amplitude is zero and the phase is indeterminate. There exist some ways to study such points, in particular when they are displaced and form an asymmetric field. The simplest method is the phase map of the relative phase (σ), where at each vortex core we find a phase singularity For a binary system like exciton-polariton, the vortex cores are available in each mode (upper and lower), and the phase map of relative phase represents double singularities, each corresponds to the vortex core in one mode. An example is shown in Figure S2 and also in Figure 3(a) of the main text. We note that in the eigenstate basis, the vortex cores are stationary (relative to the Rabi cycles, i.e., apart from the slow diffusive changes), while in superposition states they move, because Rabi coupling is beating between the photon and exciton fields.

Another way to consider displaced vortices in our binary system is through relative amplitude s. Indeed the displacement of vortices results in instantaneous change of amplitudes and phases of associated fields at each point of space, and as a consequence, the displaced polaritonic vortices can be characterized by space varying s and σ; the details of such analysis can be found in our previous publications [5, 7]. Space chart of s is shown in panel (b) in Figure S2. Interestingly s is not homogeneous in space, and one can find all quantum states simultaneously present in space (corresponding to the simultaneous presence in space of all the combinations of the (s, σ) coordinates of the sphere). Mathematically, all combinations of $-1 \leq s \leq 1$ and $0 \leq \sigma \leq 2\pi$ are present simultaneously. Corresponding isolines are shown in panel (d). The conspicuous features are: (1) that the relative phase isolines come together radially at available singularities; (2) and that $\max[s] = 1$ and $\min[s] = -1$ isodensity are positioned at the vortex core positions. Moreover, these isolines may show new topological aspects, such as saddle points (in this fundamental example, these are moved to infinity, while in the case of the main text they are represented in the plot). Another useful representation, which is common in polarization optics, is based on the corresponding ellipses defined for each quantum sate. Such elliptical fields can be used to show the peculiar property of displaced vortices in binary fields, where we can find ellipses of different shapes and orientations in space. Important features are: (1) at vortex cores of normal modes the state of pseudo-polarization is circular. (2) at each point along the trajectory of bare modes vortices, the state of pseudo-polarization in linear. Such features are shown in panel (d) in Figure S2. So far we considered some properties of quantum states in normal bases. We discuses in the introduction (main text) that upper and lower polaritons are indeed the superposition states. Any incoherent process (such as decay or scattering) may destroy such superposition giving rise to vanishing the polariton states, that is, after destroying the superposition it falls to one of the basis states (here exciton and photon) that form the superposition. In the case of exciton polariton, the photons can leave the microcavity, and

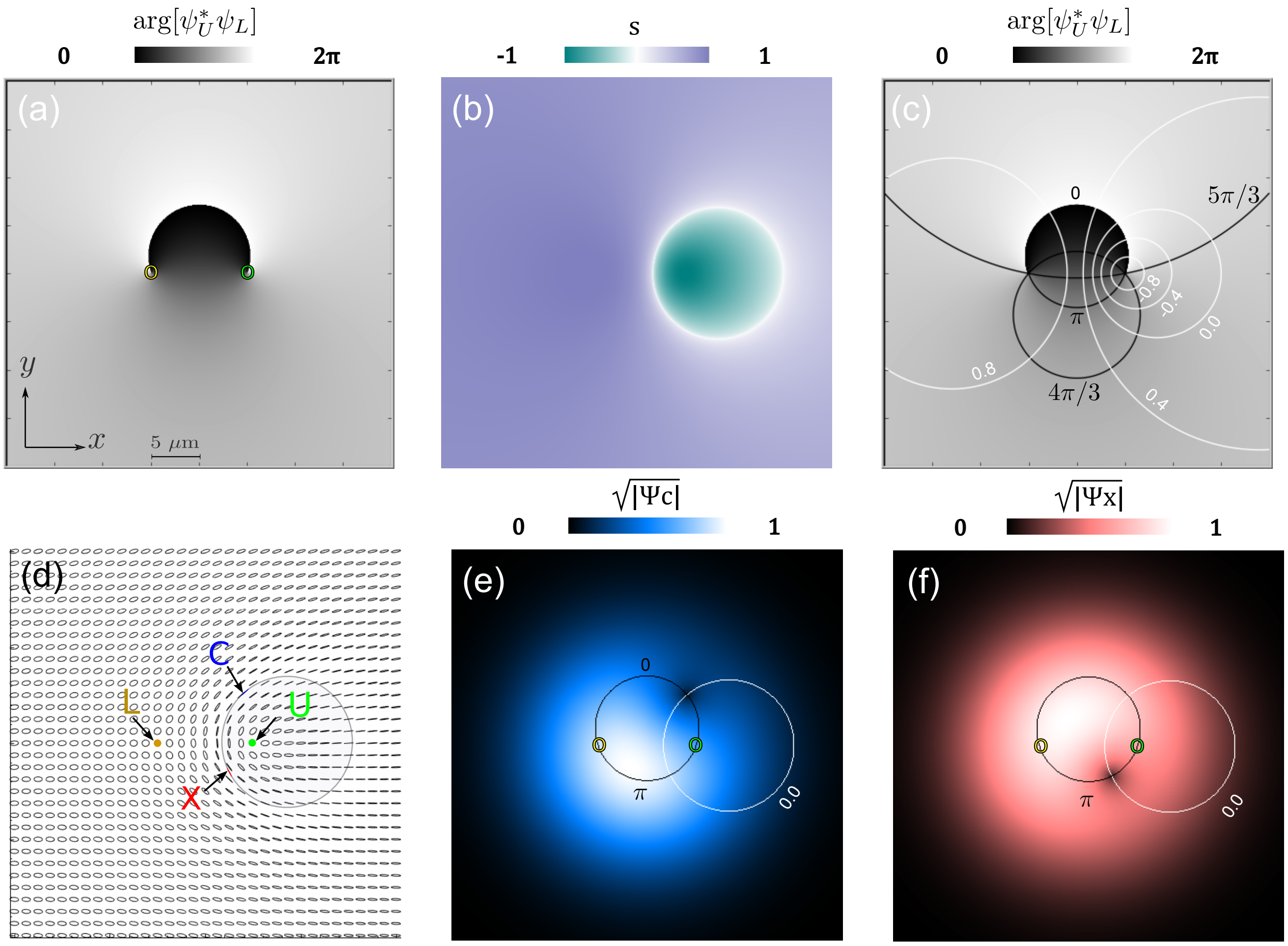

Figure S2. Various representations of the fields morphology, namely, based on their relative phase σ and amplitude s (in upper-lower polariton basis), isolines of σ, ellipses together with vortex cores, and density (absolute value) of the photon and exciton fields. (a) The phase map of relative phase, which also shows the positions of vortices in the upper-lower basis. Indeed, the unitary vortex charges in each of the two fields, compose a vortex dipole in the relative phase map. (b) The relative amplitude of the quantum states which is given by s, varies in space. The isodensity $s = 0$, here is a circle, gives the trajectory of vortex cores in photon and exciton field. (c) Isolines of the relative phase, shown as black curves, come together to the position of vortex cores. The isolines of the relative amplitudes, shown as white curves, are 1 or -1 on C,X vortex cores trajectories. (d): Elliptical representation of the polariton states and their spatial variations in the 2D space. The green (yellow) point, where the state of ellipse becomes a circle, corresponds to the upper (lower) vortex core. The state of ellipse is linear at the exciton and photon vortex cores (blue diagonal and red anti-diagonal, respectively). Vortex cores in the photon-exciton basis move along a gray trajectory, corresponds $s = 0$ in (b). All quantum states are simultaneously present in 2D. (e) The amplitude (density) map of the photon field, with the position of the vortex core at intersection of $s = 0$ and $\sigma = 0$. (f) The amplitude map of the exciton field, with the position of the vortex core at intersection of $s = 0$ and $\sigma = \pi$. The vortex cores in real space move along the white curve.

are detected via some suitable devices. It is natural to ask what we have in photon-exciton basis. Examples are shown in panels (e) and (f), in Figure S2. The density map is asymmetric in each field, and the C,X vortex cores move along an orbit corresponds to the isodensity $s = 0$.

IV. DESCRIPTION OF SUPPLEMENTARY MOVIES

SM1: Animation movie for the Photon Density Profile $|\psi_C|^2$.

SM2: Animation movie for the Exciton Density Profile $|\psi_X|^2$.

SM3: Animation movie for the Lower Polartion Density Profile $|\psi_L|^2$.

SM4: Animation movie for the Upper Polartion Density Profile $|\psi_U|^2$.

SM5: Animation Movie for a Slice Near the Max of the Photon Density.

---

[1] N. Takemura, M. D. Anderson, S. Biswas, M. Navadeh-Toupchi, D. Y. Oberli, M. T. Portella-Oberli, and B. Deveaud, "Coherent and incoherent aspects of polariton dynamics in semiconductor microcavities," Phys. Rev. B **94**, 195301 (2016).

[2] D. Sanvitto, F. M. Marchetti, M. H. Szymańska, G. Tosi, M. Baudisch, F. P. Laussy, D. N. Krizhanovskii, M. S. Skolnick, L. Marrucci, A. Lemaître, J. Bloch, C. Tejedor, and L. Viña, "Persistent currents and quantized vortices in a polariton superfluid," Nat. Phys. **6**, 527–533 (2010).

[3] T. C. H. Liew, Yuri G. Rubo, and A. V. Kavokin, "Generation and dynamics of vortex lattices in coherent exciton-polariton fields," Phys. Rev. Lett. **101**, 187401 (2008).

[4] Lorenzo Dominici, David Colas, Antonio Gianfrate, Amir Rahmani, Vincenzo Ardizzone, Dario Ballarini, Milena De Giorgi, Giuseppe Gigli, Fabrice P. Laussy, Daniele Sanvitto, and Nina Voronova, "Full-Bloch beams and ultrafast Rabi-rotating vortices," Phys. Rev. Res. **3**, 013007 (2021).

[5] Fazele Hosseini, Mohammad A. Sadeghzadeh, Amir Rahmani, Fabrice P. Laussy, and Lorenzo Dominici, "Temporal shaping and time-varying orbital angular momentum of displaced vortices," Optica **7**, 1359–1371 (2020).

[6] B. E. A. Saleh and M. C. Teich, *Fundamental of Photonics* (Wiley; 2nd edition, 2007).

[7] Lorenzo Dominici, Nina Voronova, David Colas, Antonio Gianfrate, Amir Rahmani, Vincenzo Ardizzone, Dario Ballarini, Milena De Giorgi, Giuseppe Gigli, Fabrice P. Laussy, and Daniele Sanvitto, "Shaping the topology of light with a moving rabi-oscillating vortex," Opt. Express **29**, 37262–37280 (2021).
